# Tracing the evolutionary origins of antiviral immunity

**DOI:** 10.1371/journal.pbio.3002481

**Published:** 2024-02-06

**Authors:** James B. Eaglesham, Philip J. Kranzusch

**Affiliations:** 1 New England Biolabs, Ipswich, Massachusetts, United States of America; 2 Department of Microbiology, Harvard Medical School, Boston, Massachusetts, United States of America; 3 Department of Cancer Immunology and Virology, Dana-Farber Cancer Institute, Boston, Massachusetts, United States of America

## Abstract

Animal and bacterial cells use shared mechanisms to defend against viruses. This Primer explores a PLOS Biology study which uses analysis of three families of immune genes to illuminate this evolutionary connection and trace the emergence of antiviral signaling across domains of life.

## Teaser

Animal and bacterial cells use shared mechanisms to defend against viruses. Analyzing 3 families of immune genes, a new study in *PLOS Biology* illuminates this evolutionary connection and traces the emergence of antiviral signaling across domains of life.

**Fig 1:**
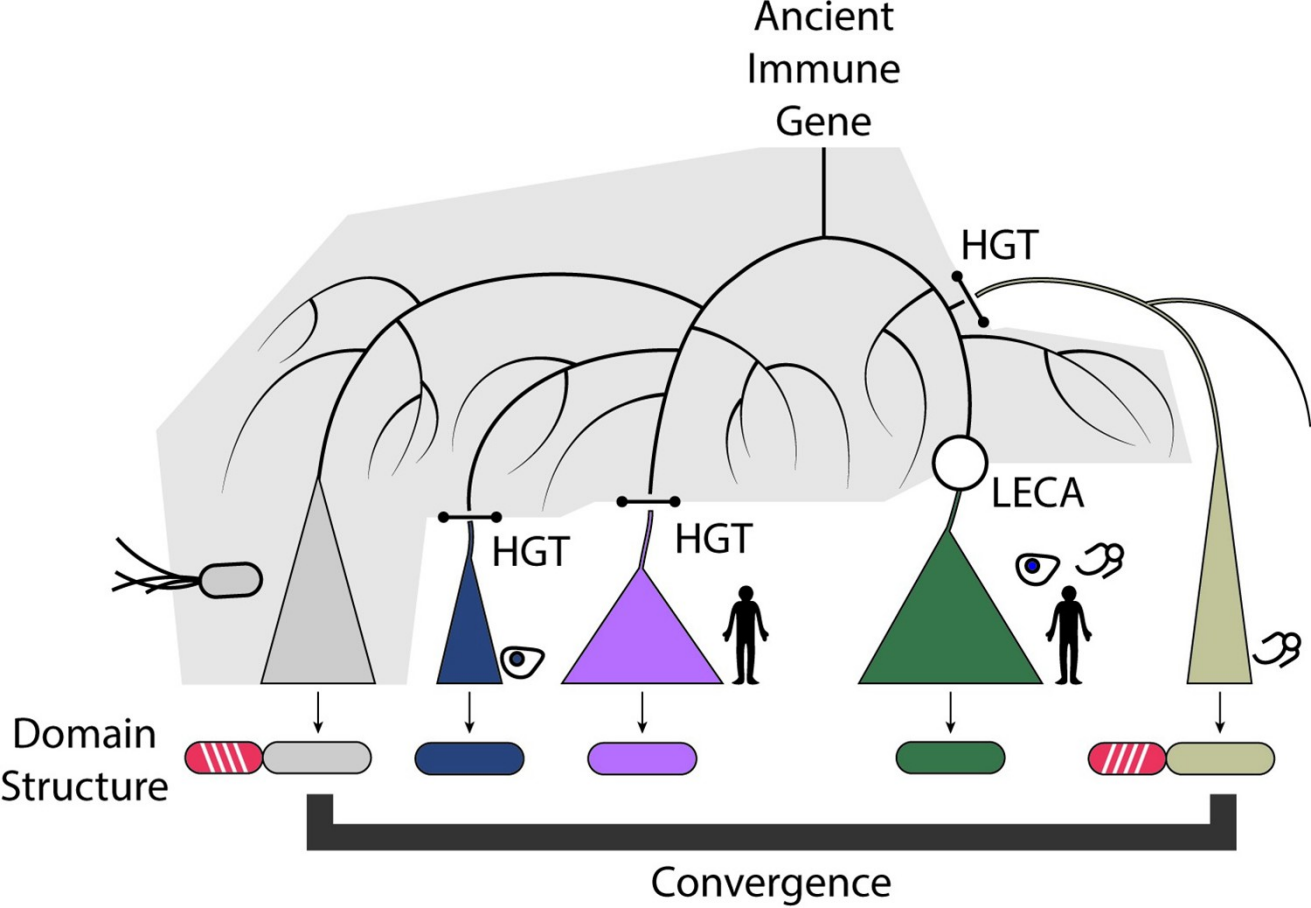
Evolutionary routes connecting ancient immune genes to modern eukaryotic genomes. Illustration of evolutionary mechanisms enabling eukaryotic acquisition of innate immune machinery from bacteria. Gray background illustrates bacterial sequences, with transition to white background indicating inheritance by eukaryotes. Through analysis of the evolutionary history of CD-NTase, STING, and viperin proteins, Culbertson and Levin highlight 3 broad mechanisms of eukaryotic immune gene evolution: vertical inheritance from LECA (green clade), horizontal gene transfer to specific eukaryotic clades groups (blue, pink, and tan clades), and convergent domain shuffling where similar accessory domains (red with white hashes) are found fused to the same immune gene (tan clade). CD-NTase, cGAS/DncV-like nucleotidyltransferase; HGT, horizontal gene transfer; LECA, last eukaryotic common ancestor; STING, stimulator of interferon genes.

Recent discoveries characterizing bacterial antiphage defense systems have generated a surprising observation: Bacteria and human cells use strikingly similar mechanisms to protect against viral infection [1,2]. As founding examples, key components of human immunity including cyclic GMP-AMP synthase (cGAS), stimulator of interferon genes (STING), and viperin each originated in ancient antiviral systems of bacteria and archaea [3–6]. In animal cells, cGAS and other cGLRs (cGAS-like receptors) sense foreign nucleic acid and catalyze synthesis of nucleotide immune signals including 2′3′-cGAMP (2′–5′/3′–5′ cyclic GMP–AMP). 2′3′-cGAMP activates the downstream receptor STING and induces antiviral responses through type I interferon and NF-κB signaling [2,7]. Type I interferons can induce expression of a suite of antiviral effector proteins including the enzyme viperin that creates chain-terminating nucleotide analogs that potently inhibit viral transcription and genome replication [6]. With the realization that central aspects of human immunity are shared with bacteria, a key question is how did this come to be? In this issue of *PLOS Biology*, Culbertson and Levin provide a comprehensive bioinformatic analysis of eukaryotic cGLR, STING, and viperin proteins, revealing strikingly different evolutionary histories and providing insight into the early emergence of metazoan antiviral immunity (Fig 1) [8].

To explain evolution of antiviral immunity, Culbertson and Levin design and iterate a sensitive search strategy to uncover divergent homologs of cGLR, STING, and viperin proteins. Leveraging EukProt, a curated database representative of eukaryotic protein diversity [9], the authors designed a conservative sequence homology search strategy to create a comprehensive set of eukaryotic immune proteins for direct comparison with components of bacterial antiphage defense. First, the authors compare animal cGLRs with bacterial cGAS-like enzymes named CD-NTases (cGAS/DncV-like nucleotidyltransferases) [3]. Surprisingly, cross-domain phylogenetic analysis suggests multiple pathways through which *CD-NTase* genes entered eukaryotic genomes and seeded animal immune protein families. Distinct phylogenetic clades include cGLRs (including the human DNA sensor cGAS), oligoadenylate synthase (OAS)-like enzymes (including the human double-stranded RNA sensor oligoadenylate synthase 1), and a set of uncharacterized proteins the authors name “eSMODS” after the protein domain name “second messenger oligo- or di-nucleotide synthase” [10]. OAS-family proteins are broadly present among eukaryotes and form a monophyletic, well-supported clade indicating presence at LECA (last eukaryotic common ancestor). In contrast, cGLR and eSMODS proteins appear to have evolved later via independent horizontal gene transfers of bacterial CD-NTases. cGLRs are encoded mainly in metazoans, where the authors demonstrate further diversification and gene duplication, and eSMODS appear in a subset of microeukaryotes.

Using a similar strategy to analyze STING proteins, the authors uncover 2 instances of convergent domain shuffling, which shaped evolution of this gene family. In bacteria, STING antiphage defense proteins are encoded either with a 2-pass transmembrane (TM) or a toll-interleukin receptor (TIR) effector domain [5]. In contrast, animal STING proteins have a 4-pass TM domain, which, in mammals, is required for cellular trafficking and signaling. Interestingly, the authors uncovered several bacteria-like STING homologs, mainly in unicellular eukaryotes, which exhibit closer homology to bacterial STINGs than mammalian STINGs in the cyclic dinucleotide ligand binding domain. However, these bacteria-like STINGs are fused to a 4-pass TM domain similar to mammalian STINGs. Further, some eukaryotic STINGs acquired a TIR-STING architecture via fusion with a eukaryotic TIR supporting convergent evolution where bacterial and animal STINGs independently acquired similar domain fusions [10].

Finally, analyzing viperin evolution, the authors demonstrate that most eukaryotic viperin homologs form a single well-supported phylogenetic clade and that viperin enzymes likely only entered animals once via descendance from LECA. However, Culbertson and Levin also identify 2 groups of marine algal viperin homologs, which branch from marine cyanobacterial viperin proteins within the bacterial section of the phylogenetic tree. This observation may support additional horizontal gene transfer events in viperin evolutionary history and suggests that eukaryotes continue to sample bacterial pools of antiviral genes.

In addition to these exciting observations, the authors note some current limitations of their bioinformatic approach. Eukaryotic genome assemblies and transcriptomes are of varying qualities and frequently contain sequence contamination from prokaryotes, complicating interpretations of horizontal gene transfer between domains. The authors are careful to only propose apparent horizontal gene transfer events if a well-supported monophyletic group of several eukaryotic sequences branched from a bacterial sequence clade. They also note that this approach is unable to distinguish prokaryotic endosymbionts and/or close prokaryotic–eukaryotic cell associations, which may repeatably result in contamination of genome assemblies with similar sequences. This may be a particular issue among microeukaryotes, supporting a need for more high-quality genome assemblies and establishment of biological models to investigate the nature of immunity in these organisms.

Culbertson and Levin hint at major developments still to come in our understanding of the evolution and biology of antiviral immunity in eukaryotes. Domain-wide screens such as those which have characterized CD-NTases, cGLRs, and viperin proteins [3,6,7] will be of use to better understand the molecular function of the newly identified eSMODS, blSTING, and algal viperin proteins. Additionally, the distinct paths of cGLR/CD-NTase and STING protein evolution observed in this study indicates that these proteins may have alternative functions either individually or within different pathways in non-metazoans. Finally, the authors’ observation that a majority of species currently within the EukProt database lack identifiable homologs of these 3 major immune proteins (711/933) may indicate adoption of alternative immune strategies in many species. Given the deep reservoir of bacterial antiphage defense system genes from which eukaryotes sample immune functions, further analysis of non-metazoan eukaryotes represents a new frontier to understand antiviral immunity and host–virus interactions across all domains of life.

## Abbreviations

CD-NTase: cGAS/DncV-like nucleotidyltransferase
cGAS: cyclic GMP-AMP synthase
cGLR: cGAS-like receptor
LECA: last eukaryotic common ancestor
OAS: oligoadenylate synthase
STING: stimulator of interferon genes
TIR: toll-interleukin receptor
TM: transmembrane
2′3′-cGAMP: 2′–5′/3′–5′ cyclic GMP–AMP
